# Knockdown of long non-coding RNA SOX2OT downregulates SOX2 to improve hippocampal neurogenesis and cognitive function in a mouse model of sepsis-associated encephalopathy

**DOI:** 10.1186/s12974-020-01970-7

**Published:** 2020-10-25

**Authors:** Jialin Yin, Yanan Shen, Yanna Si, Yuan Zhang, Jiayue Du, Xiajuan Hu, Mengmeng Cai, Hongguang Bao, Yan Xing

**Affiliations:** 1 Department of Anesthesiology, Nanjing First Hospital, Nanjing Medical University, Nanjing, 210006 People’s Republic of China; 2 Mechanical Engineering, Southeast University, Nanjing, 211118 People’s Republic of China

**Keywords:** Sepsis-associated encephalopathy, Cognitive dysfunction, Hippocampal neurogenesis, SOX2OT, SOX2

## Abstract

**Background:**

Aberrant hippocampal neurogenesis is an important pathological feature of sepsis-associated encephalopathy. In the current study, we examined the potential role of the long noncoding RNA (lncRNA) sex-determining region Y-box 2 (SOX2) overlapping transcript (SOX2OT), a known regulator of adult neurogenesis in sepsis-induced deficits in hippocampal neurogenesis and cognitive function.

**Methods:**

Sepsis was induced in adult C57BL/6 J male mice by cecal ligation and perforation (CLP) surgery. Randomly selected CLP mice were transfected with short interfering RNAs (siRNAs) against SOX2OT or SOX2, or with scrambled control siRNA. Cognitive behavior was tested 8–12 days post-surgery using a Morris water maze. Western blotting and RT-qPCR were used to determine expression of SOX2, Ki67, doublecortin (DCX), nestin, brain lipid-binding protein, and glial fibrillary acidic protein (GFAP) in the hippocampus. The number of bromodeoxyuridine (BrdU)^+^/DCX^+^ cells, BrdU^+^/neuronal nuclei (NeuN)^+^ neurons, and BrdU^+^/GFAP^+^ glial cells in the dentate gyrus were assessed by immunofluorescence.

**Results:**

CLP mice showed progressive increases in SOX2OT and SOX2 mRNA levels on days 3, 7, and 14 after CLP surgery, accompanied by impaired cognitive function. Sepsis led to decrease in all neuronal markers in the hippocampus, except GFAP. Immunofluorescence confirmed the decreased numbers of BrdU^+^/DCX^+^ cells and BrdU^+^/NeuN^+^ neurons, and increased numbers of BrdU^+^/GFAP^+^ cells. SOX2OT knockdown partially inhibited the effects of CLP on levels of SOX2 and neuronal markers, neuronal populations in the hippocampus, and cognitive function. SOX2 deficiency recapitulated the effects of SOX2OT knockdown.

**Conclusion:**

SOX2OT knockdown improves sepsis-induced deficits in hippocampal neurogenesis and cognitive function by downregulating SOX2 in mice. Inhibiting SOX2OT/SOX2 signaling may be effective for treating or preventing neurodegeneration in sepsis-associated encephalopathy.

## Background

Sepsis is a primary clinical challenge and often results in multiple organ dysfunction. Sepsis and its complications are the most frequent cause of high morbidity and mortality in the intensive care unit [1, 2], although recent medical advances have substantially enhanced survival rates. Sepsis-associated encephalopathy is a severe complication with incidence of 9–71%. Patients with sepsis-associated encephalopathy show various degrees of cognitive dysfunction, ranging from impaired consciousness to delirium that can persist for months to years after hospital discharge. These patients often require long-term medical interventions in order to regain function [2]. These adverse neurobehavioral consequences are often associated with reduced quality of life, poor prognosis, and increased morbidity and mortality [3].

The cognitive impairment in sepsis-associated encephalopathy is associated with dysfunctional hippocampal neurogenesis [4]. Neural stem cells persist into adulthood and maintain the production, survival, migration, and recruitment of new neurons [5, 6], but studies in a mouse model of sepsis induced by lipopolysaccharide indicate decreased proliferation and survival of new neurons, and dysfunction differentiation in the dentate gyrus [4, 7]. These animals also show decreased synaptic plasticity, leading to learning and memory deficits [4, 7]. Brain neurogenesis has been shown to be regulated by long noncoding RNAs (lncRNAs), a class of transcripts longer than 200 nucleotides [8–10]. Therefore, treatments based on lncRNA may be effective against neurodegeneration induced by sepsis-associated encephalopathy [11].

Sex-determining region Y-box 2 (SOX2) overlapping transcript (SOX2OT), a highly conserved lncRNA among vertebrates [12], has been linked to cognitive impairment in a mouse model of Alzheimer’s disease [13]. SOX2OT is expressed in zones of active neurogenesis in adult mouse brain [14]. SOX2OT is also highly expressed in areas of dynamic proliferation and differentiation of adult neural stem cells [12]. SOX2 arises from a single exon within one of the SOX2OT introns and the two transcripts are frequently detected together, perhaps due to their overlapping transcriptional orientation [12, 13, 15]. An in vitro study involving neuronal stem and progenitor cells isolated from the subventricular zone of adult mice showed that SOX2OT and SOX2 are upregulated in the early stages of neuronal proliferation; then, their levels progressively decrease as cells differentiate [12]. In fact, SOX2OT downregulates SOX2 in neural progenitor cells and thereby represses neural progenitor proliferation and promotes cortical neurogenesis in the developing mouse brain [15].

Therefore, we sought to examine here whether lncRNA SOX2OT may be involved in sepsis-induced hippocampal neurogenesis and cognitive dysfunction in a mouse model. We also asked whether knocking down SOX2OT may ameliorate these sepsis-induced effects by downregulating SOX2.

## Material and methods

### Animals

All animal experimental protocols were approved by the Institutional Animal Care and Use Committee at Nanjing Medical University (protocol reference number 2017-142). Adult male C57BL/6 J mice (6–8-week old, 25–31 g, Experimental Animal Center at Nanjing First Hospital) were housed in a temperature- and humidity-controlled facility on a 12-h light-dark cycle with free access to food and water. Only male mice were used in the experiments to minimize the heterogeneity due to sex differences in the pathology of encephalopathy [16].

### Experimental design

**Fig. 1:**
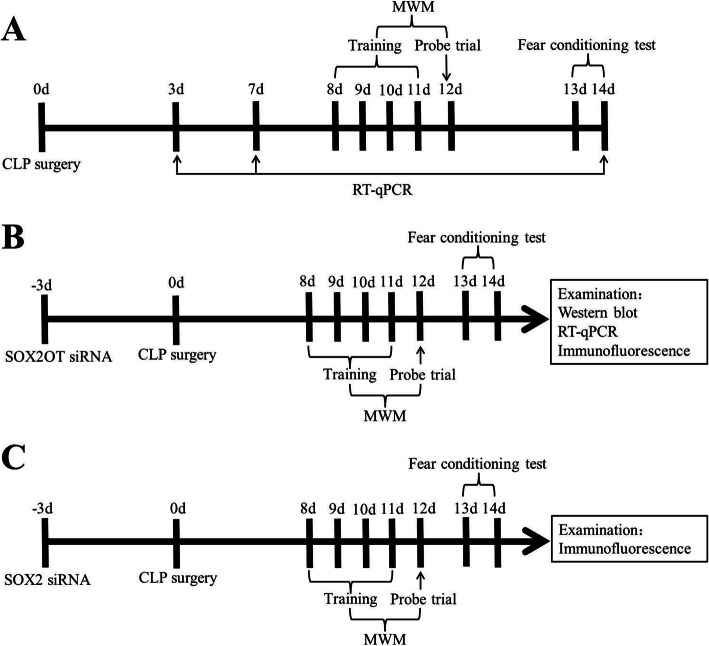
Schematic illustration of the experimental design. **a** Timeline for naive, sham-operated, or CLP-operated mice. **b** Timeline for mice injected with scrambled control siRNA or SOX2OT siRNA. **c** Timeline for mice injected with scrambled control siRNA or SOX2 siRNA. Abbreviations: CLP, cecal ligation and perforation; d, day; MWM, Morris water maze; RT-qPCR, reverse transcription-quantitative polymerase chain reaction; siRNA, short interfering RNA; SOX2, sex-determining region Y-box 2; SOX2OT, SOX2 overlapping transcript

The mouse sepsis model was established using cecal ligation and perforation (CLP) surgery as previously described [16]. Mice were randomly assigned to the following groups (*n* = 15/group): naïve, sham operated, or CLP. CLP mice were further treated with one of the following (*n* = 16/group): short interfering RNA (siRNA) against SOX2OT, siRNA against SOX2, or scrambled control siRNA 3d before CLP. After CLP surgery, mice were subjected to the Morris water maze trial for 5 days starting on day 8, and a fear conditioning test was performed on day 13 (Fig. 1). Mice were sacrificed, and the hippocampus was removed for RT-PCR, western blotting, and immunohistochemistry.

### CLP surgery

**Fig. 2:**
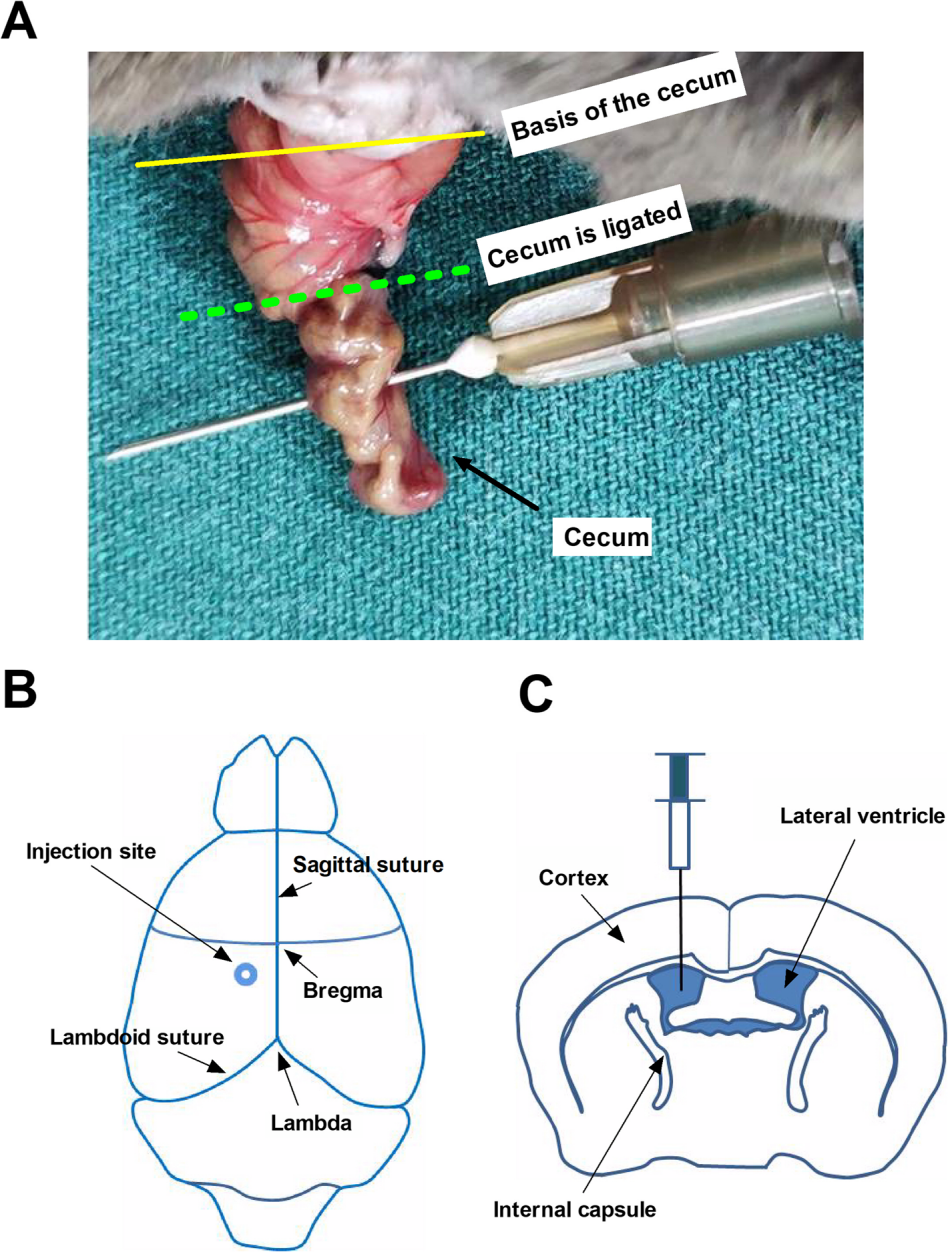
A schematic illustration of CLP procedure and i.c.v. injection. **a** CLP procedure. The basis of the cecum is indicated by the yellow line. The cecum is ligated (indicated by dotted green line) at half the distance between distal pole and the base of the cecum. **b** Injection site of the mouse lateral ventricle. The injection site is at 1.0 mm lateral and 0.5 mm posterior to the bregma over the left hemisphere. **c** A 27 G needle with a 10-μl syringe is used for the injection into the lateral ventricle 2.5-mm deep, perpendicular to the skull surface. Abbreviations: CLP, cecal ligation and perforation; i.c.v., intracerebroventricular

The mouse sepsis model was established as previously described [16] with some modifications. Briefly, animals were anesthetized using intraperitoneal ketamine (80 mg/kg) and xylazine (5 mg/kg). After disinfection, a 2–3-cm incision was made lengthwise below the xiphoid to expose the abdominal cavity. The cecum was isolated and ligated at half the distance between distal pole and the base of the cecum, and punctured twice with a 22-gauge needle (Fig. 2a). Fecal contents were gently extruded into the peritoneal cavity. Then, the cecum was carefully placed back into the abdominal cavity, and the abdomen was sutured. For sham-operated animals, the abdominal cavity was opened to expose the cecum without ligation or perforation. After surgery, 1 ml of sterile saline prewarmed to 37 °C was given intraperitoneally for rehydration. Animals were returned to their cages with a warm cotton pad and free access to food and water. Naïve mice did not undergo laparotomy.

### Bromodeoxyuridine labeling

Bromodeoxyuridine (BrdU) is an S-phase marker of proliferating cells. To identify newly synthesized DNA after CLP surgery, animals received BrdU (50 mg/kg; Sigma, St. Louis, MO, USA) via intraperitoneal injection twice daily at 8 h intervals for seven consecutive days before CLP.

### SOX2OT and SOX2 knockdown in vivo

SOX2OT and SOX2 were knocked down using siRNA with the following sequence: 5′-CAG CAG AGA AAT GAT ATC TAG ACC A-3′ for SOX2OT (scrambled control sequence: 5′-GAC CGA TAA CGA AAG CAC AAA TTG T-3′) and 5′-GCG GCG ACC GGC GGC AAC CAG AAG A-3′ for SOX2 (scrambled control: 5′-GAG AGC ACC GGG ACG CCG AGA CAG C-3′). The siRNAs were designed and synthesized by GenePharma (Shanghai, China). RNAs were transfected using Entranster^TM^ in vivo transfection reagent (Engreen Biosystem, Beijing, China). The Entranster^TM^-in-vivo-siRNA mixture was performed according to the manufacturer’s instructions as previously described [17]. Briefly, 5 μg of siRNA was dissolved in 5 μL RNase-free water, then gently mixed with 5 μL of in vivo transfection reagent. The mixture was kept at room temperature for 15 min, then injected intracerebroventricularly (i.c.v*.*) using a stereotaxic apparatus as previously described [18]. The stereotaxic coordinates were 1.0 mm lateral and 0.5 mm posterior to the bregma over the left hemisphere, and 2.5 mm below the dural surface (Fig. 2b/c). SOX2OT or SOX2 knockdown was verified using RT-PCR or Western blot.

### Morris water maze

A standard 5-day Morris water maze test was performed by a researcher blinded to treatment groups [19]. The water maze consisted of a round, painted pool with a diameter of 100 cm and height of 40 cm. The pool was filled with opaque water to a depth of 28 cm and maintained at 23 ± 1 °C. The initial 4 days included spatial acquisition training and testing sessions, which included four sessions per day at an inter-session interval of 20 min. The maximum length of the sessions was 60 s. Animals were trained to escape by swimming onto a hidden platform at 0.5 cm beneath the water surface in the original target quadrant. Animals that failed to locate the platform within 60 s were manually guided to the platform. Mice were allowed to stay on the platform for 20 s before being removed. Swimming speed and escape latency were analyzed using a motion detection software (Actimetrics Software, Evanston, IL, USA). The escape latency from all four sessions was averaged to calculate the escape latency for each day. On the fifth day, the platform was removed to allow for probe testing. During the 60-s session, the number of crossings over the target quadrant and the total time spent in that quadrant were recorded.

### Fear conditioning test

A fear conditioning testing was conducted the day after the Morris water maze experiments were completed [20]. All mice were placed in a 70% ethanol-wiped chamber with a floor of stainless-steel bars. This chamber was designed to allow sound stimulation for the training of conditioned reflexes, as well as electrical stimulation on the feet using a constant-current generator. Mice were allowed to explore and habituate in the training environment for 3 min. Then, they were stimulated with a monofrequency sound for 30 s (1 kHz, 80 dB), and during the last 2 s of this sound, a foot shock (1 mA) was delivered. After a 30-s pause, this pairing of sound and shock was delivered a second time. After 24 h, as a test of contextual memory, mice were allowed to enter the original chamber without any stimulation to monitor freezing behavior, defined as the absence of any movement other than that required for respiration for more than 3 s. Then, 2 h later, a cued fear conditioning test was conducted in which the animals were placed in the chamber and subjected to the monofrequency sound for 3 min without foot shock, and freezing behavior was monitored and recorded.

### Western blotting

Hippocampal tissues were homogenized and centrifuged at 13,000 *g* at 4 °C for 10 min. Proteins in the supernatant were separated by sodium dodecyl sulfate–polyacrylamide gel electrophoresis (SDS-PAGE). Proteins were then transferred onto a nitrocellulose membrane (Hybond-ECL, Amersham Biosciences, Little Chalfont, UK). Membranes were blocked and incubated overnight at 4 °C with primary antibodies against octamer-binding transcription factor 4 (OCT4; 1:200), doublecortin (DCX; 1:1000), or β-actin (1:200) (all from Santa Cruz Biotechnology, Santa Cruz, CA, USA); against SOX2 (1:200), Ki67 (1:1000), nestin (1:1000), or brain lipid-binding protein (BLBP; 1:800) (all from Abcam, Cambridge, MA, USA); or against glial fibrillary acidic protein (GFAP; 1:1000; EMD Millipore, Billerica, MA, USA). After thorough washing, blots were incubated with secondary antibodies (Sigma-Aldrich, St. Louis, MO, USA) conjugated with horseradish peroxidase. Protein bands were detected using enhanced chemiluminescence (Amersham Biosciences) and quantitated using the Quantity One software (Bio-Rad Laboratories, Hercules, CA, USA). Band intensities were normalized to β-actin.

### Reverse transcription-quantitative polymerase chain reaction analysis

Total RNA was extracted from hippocampal tissue using Trizol reagent (Invitrogen, Carlsbad, CA, USA), then used as template for single-strand cDNA synthesis in the AMV Reverse Transcriptase Kit (Promega, Madison, WI, USA). Quantitative reverse transcription-quantitative polymerase chain reaction (RT-PCR) was performed using SYBR Green I dye detection (Takara Bio, Kusatsu, Shiga, Japan) on a real-time detection system (Bio-Rad). The PCR conditions included an initial step at 95 °C for 3 min, followed by 30 cycles at 95 °C for 1 min, 56 °C for 40 s, and 72 °C for 1 min. Melting curves were created to ensure that nonspecific products were not amplified. GAPDH was used as an internal control. Relative mRNA expression of the target gene was performed by the 2^−ΔΔCT^ method. Primers for RT-qPCR are shown in Table 1 (Genepharma, Shanghai, China).

**Table 1:** Primer sequences for RT-qPCR

Primer		Primer Sequence(5′-3′)
SOX2OT	F	ACG GGC ACA CAT CAA GCA TA
	R	AGC CTC AGC CCC CTAT ACA A
SOX2	F	GGG CTC TGT GGT CAA GTC CG
	R	CGC TCT GGT AGT GCT GGG C
OCT4	F	CAA GTT GGC GTG GAG ACT TTG C
	R	CCC CAA GGT GAT CCT CTT CTG C
GAPDH	F	TGA GGC CGG TGC TGA GTA TGT CG,
	R	CCA CAG TCT TCT GGG TGG CAG TG

*F* forward, *GAPDH* glyceraldehyde 3-phosphate dehydrogenase, *OCT4* octamer-binding transcription factor 4, *R* reverse, *SOX2OT* SOX2 overlapping transcript, *SOX2* sex-determining region Y-box 2

### Immunofluorescence

Animals were perfused through the left ventricle with phosphate-buffered saline followed by 4% paraformaldehyde. After perfusion, the brain was removed, post-fixed in 4% paraformaldehyde at room temperature for 2 h, and then successively dehydrated in 20% and 30% sucrose solutions at 4 °C. Brain sections (4-μm thick) were cut in a cryostat and processed for immunofluorescence. The sections were blocked with 8% goat serum for 2 h at room temperature, then incubated overnight at 4 °C with primary antibodies against BrdU (1:200; Abcam), DCX (1:500; Abcam), glial fibrillary acidic protein (GFAP) (1:100; Abcam), and neuronal nuclei (NeuN) (1:400, Cell Signaling Technology, Beverly, MA, USA). The second antibody was anti-mouse or anti-rabbit IgG (1:1000; Cell Signaling Technology) conjugated with Alexa Fluor® 488 and Alexa Fluor® 594. The sections were counterstained with 4’,6-diamidino-2-phenylindole (DAPI) for 10 min at room temperature, and observed and imaged under an Olympus BX43 confocal microscope (Olympus, Tokyo, Japan).

### Statistical analysis

Statistical analysis was performed using the Statistical Package for Social Sciences 18.0 (IBM, Chicago, IL, USA). Data are presented as mean ± standard deviation (SD). Data from multiple groups were compared using one-way analysis of variance (ANOVA), followed by post hoc Tukey test. Data from SOX2OT and SOX2 expression patterns, and training sessions were analyzed by two-way repeated-measures ANOVA, adjusted by the Bonferroni test for main effects. Differences were considered significant when *P* < 0.05.

## Results

### Cognitive impairment and upregulation of SOX2OT and SOX2 in sepsis-associated encephalopathy

**Fig. 3:**
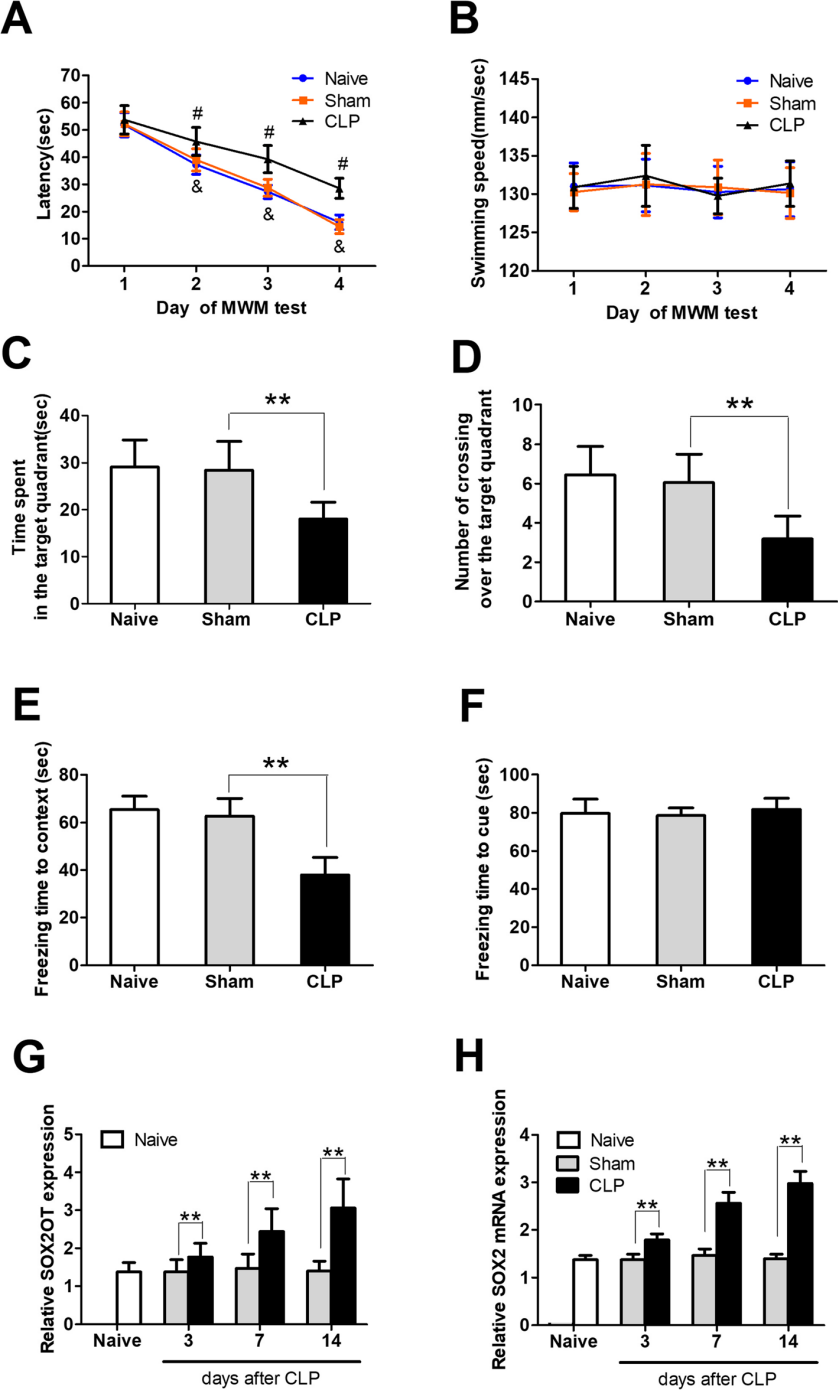
Cognitive impairment and SOX2OT and SOX2 upregulation in a mouse model of sepsis-associated encephalopathy. **a** Escape latency time, **b** swimming speed, **c** time spent in the target quadrant, and **d** number of crossings over the target quadrant in the Morris water maze. **e** Freezing time in context and **f** freezing time in response to a cue in the fear conditioning test (*n* = 15/group). Relative levels of **g** SOX2OT and **h** SOX2 mRNAs were measured by RT-qPCR on days 3, 7, and 14 after sham or CLP surgery (*n* = 5/group). ^&^*p* < 0.05 vs. day 1 of Morris water maze test; ^#^*p* < 0.05 vs. Sham; **p < 0.05. Abbreviations: CLP, cecal ligation and perforation; MWM, Morris water maze; SOX2, sex-determining region Y-box 2; SOX2OT, SOX2 overlapping transcript

In the Morris water maze, escape latency progressively decreased over the first 4 days of training in all groups (Fig. 3a). The CLP group had longer escape latencies than the sham group on days 2–4. There was no difference in swimming speed among groups (Fig. 3b), suggesting no motor dysfunction in any mice. Compared to the sham-operated group, the CLP group spent a shorter amount of time and had fewer crossings in the target quadrant on the test day (Fig. 3c and d). These results suggested that CLP mice had learning and memory deficits.

CLP mice exhibited shorter freezing time than sham mice in the contextual fear conditioning test (Fig. 3e). These results suggest that CLP mice displayed an environment-dependent impairment of the fear conditioning response. However, CLP and sham mice showed similar freezing time in the hippocampal-independent cued fear conditioning test (Fig. 3f), suggesting that the impairment was only in the hippocampus.

CLP mice showed progressively higher levels of SOX2OT transcript (Fig. 3g) and SOX2 transcript (Fig. 3h) than sham mice on days 3, 7, and 14 after CLP surgery.

### SOX2OT knockdown ameliorated sepsis-induced cognitive dysfunction in mice with sepsis-associated encephalopathy

**Fig. 4:**
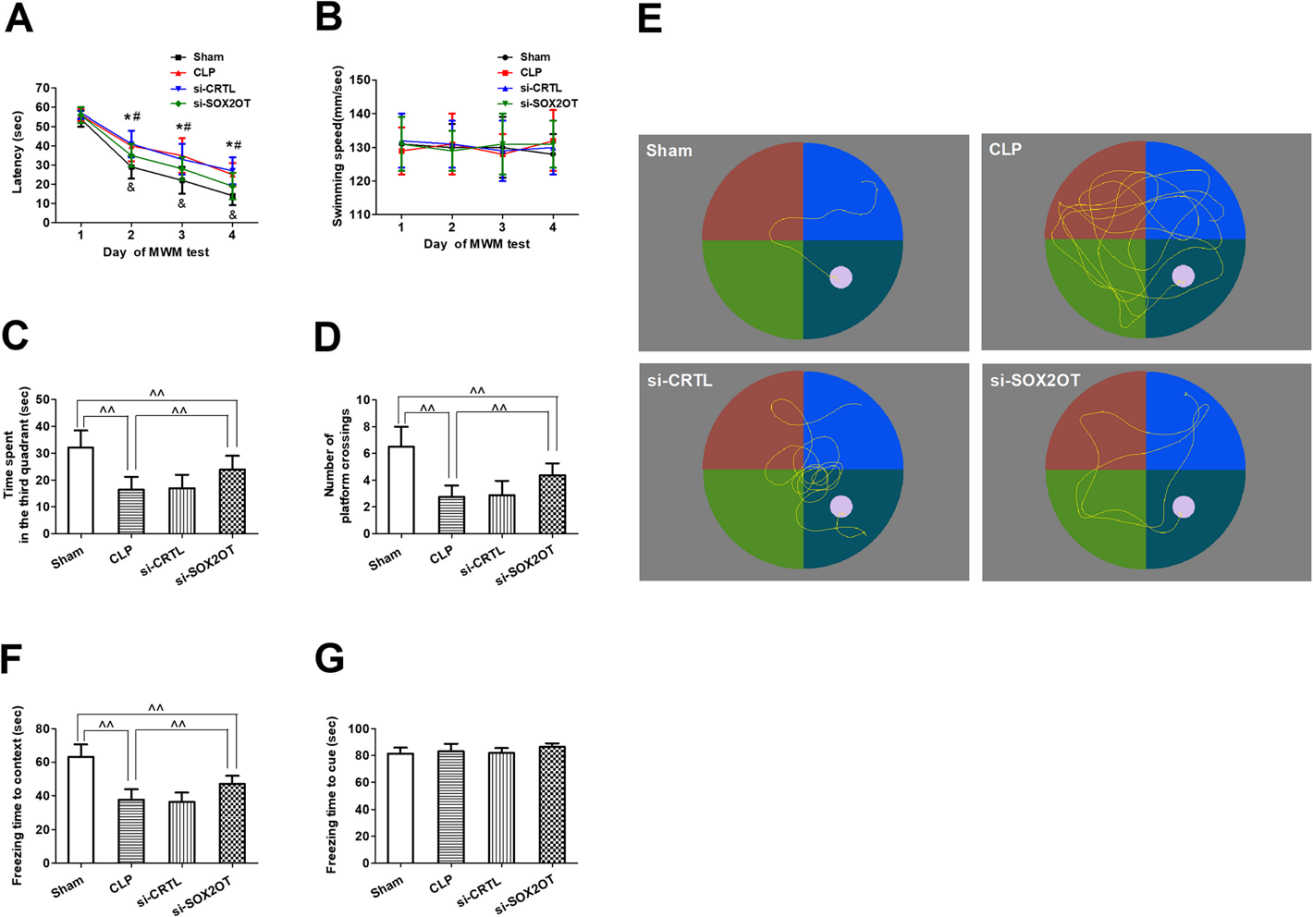
SOX2OT knockdown attenuated sepsis-induced cognitive dysfunction in mice with sepsis-associated encephalopathy. **a** Escape latency, **b** swimming speed, **c** time spent in the target quadrant, **d** number of times mice crossing the target quadrant, and **e** representative swim paths on the test day in the Morris water maze. The white circle indicates the location of the training platform, which was removed during the test. **f** Freezing time in context and **g** freezing time in response to a cue in the fear conditioning test (*n* = 16/group). ^&^*p* < 0.05 vs. day 1 of Morris water maze test; *p < 0.05 vs. Sham; ^#^*p* < 0.05 vs. CLP; ^^*p* < 0.01. Abbreviations: CLP, cecal ligation and perforation; si-CRTL, scrambled control siRNA; si-SOX2OT, SOX2OT siRNA

Since CLP mice had impaired cognitive function and upregulated SOX2OT lncRNA, which is known to regulate adult neurogenesis, we knocked down SOX2OT in vivo in order to examine whether this would mitigate the effects of CLP. In the Morris water maze, all CLP groups had longer escape latencies than the sham group on days 2–4, regardless of SOX2OT levels, but CLP mice transfected with SOX2OT siRNA had shorter escape latencies than mice with CLP alone (Fig. 4a), suggesting that SOX2OT knockdown improved learning and memory in sepsis mice. There was no difference in swimming speed between groups (Fig. 4b). Compared to sham animals, CLP mice spent less time in the target quadrant and had fewer crossings in the target quadrant on the test day (Fig. 4c–e). SOX2OT knockdown in CLP mice prolonged time in the target quadrant and led to more crossings. No significant differences were observed between CLP mice transfected with scrambled siRNA and non-transfected CLP mice.

All CLP mice had shorter freezing time in the contextual fear conditioning test than sham mice (Fig. 4f), and SOX2OT knockdown prolonged freezing time in CLP animals. These results suggest that SOX2OT knockdown protected against environment-dependent impairment of the fear conditioning response. In contrast, the groups showed no difference in freezing time in the hippocampal-independent cued fear conditioning test (Fig. 4g).

### SOX2OT knockdown partially restored cell proliferation and survival of mature neurons while reducing glial differentiation in the hippocampus of mice with sepsis-associated encephalopathy

**Fig. 5:**
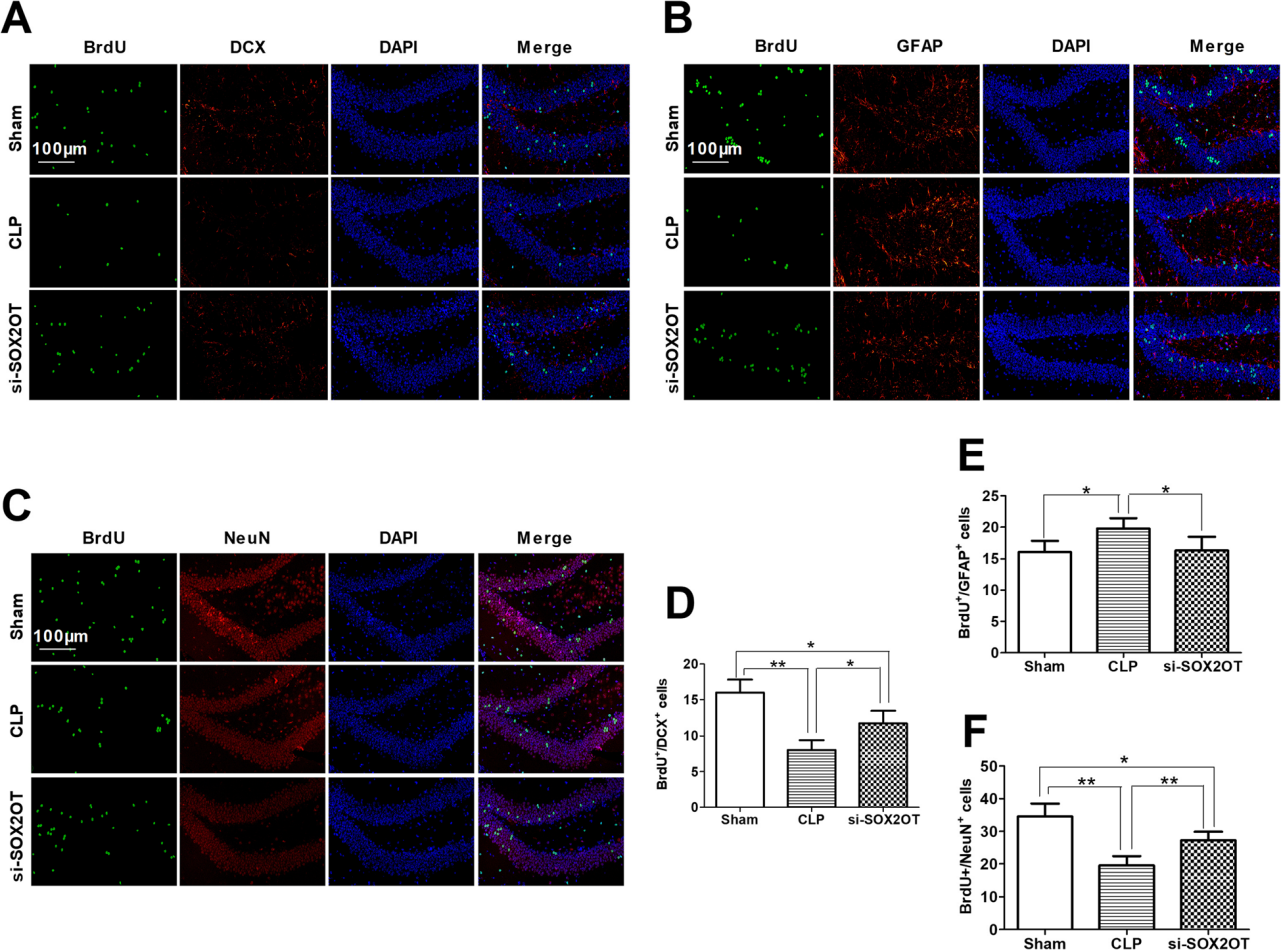
SOX2OT knockdown using siRNA attenuated the loss of cell proliferation and mature neurons, and reduced glial differentiation in the hippocampus of mice with sepsis-associated encephalopathy. Representative immunofluorescence micrographs showing (**a**) BrdU^+^/DCX^+^ cells, (**b**) BrdU^+^/GFAP^+^ glial cells and (**c**) BrdU^+^/NeuN^+^ neurons in the dentate gyrus of the hippocampus from CLP mice injected with SOX2OT siRNA. Magnification: × 200. Scale bar = 100 μm. Quantification of immunofluorescent cells staining positive for (**d**) BrdU/DCX, (**e**) BrdU/GFAP or (**f**) BrdU/NeuN. *p < 0.05; **p < 0.01. Abbreviations: CLP, cecal ligation and perforation; si-CRTL; scrambled control siRNA; si-SOX2OT, SOX2OT siRNA

CLP mice had fewer BrdU^+^/DCX^+^ cells and BrdU^+^/NeuN^+^ neurons but more BrdU^+^/GFAP^+^ glial cells in the dentate gyrus of the hippocampus than sham controls (Fig. 5). These effects of CLP were significantly inhibited by SOX2OT knockdown, although the differences remained significant between knockdown CLP animals and sham or CLP animals.

### SOX2OT knockdown attenuated changes in the hippocampal neuronal markers in mice with sepsis-associated encephalopathy

**Fig. 6:**
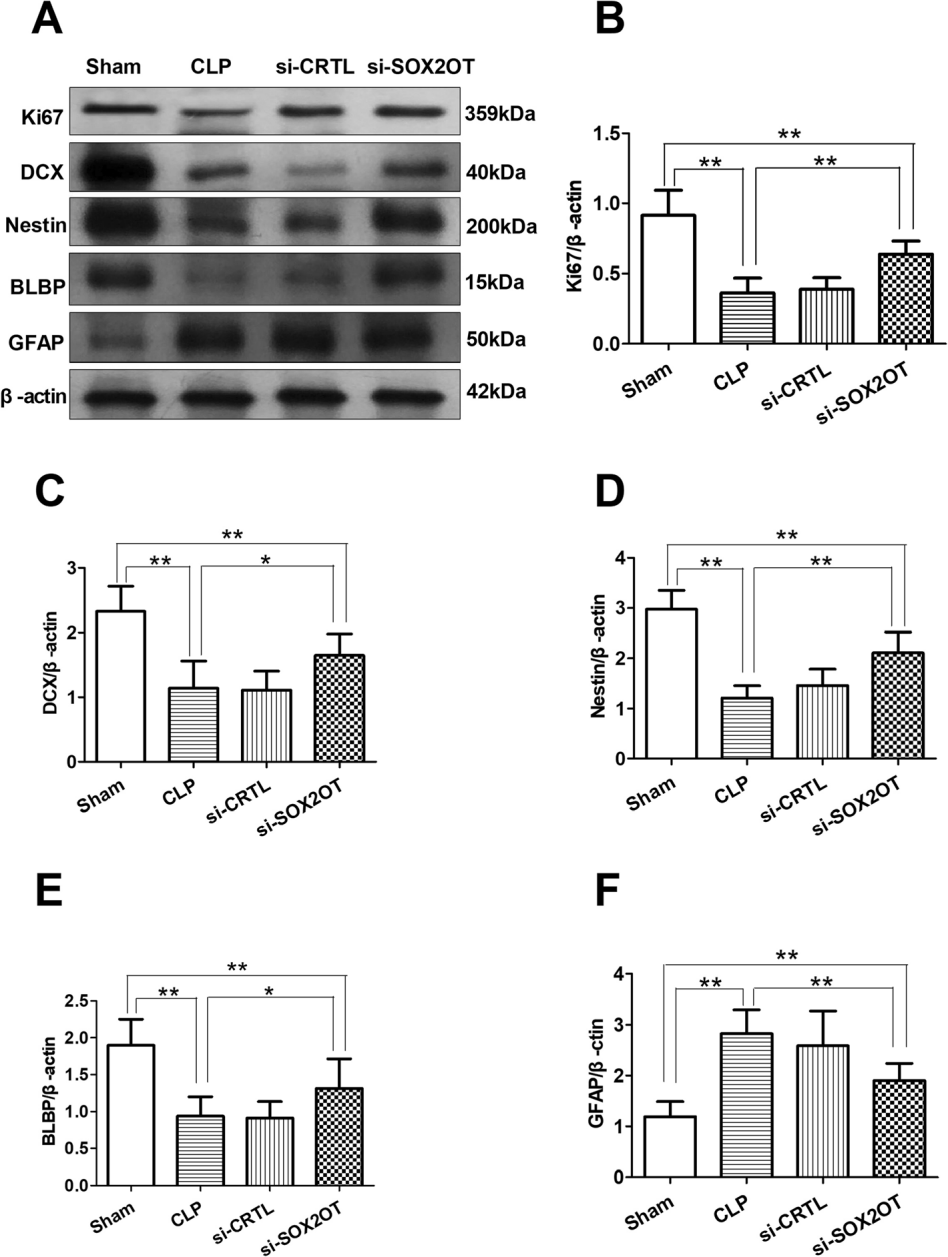
SOX2OT knockdown attenuated changes in neuronal marker levels in the hippocampus of mice with sepsis-associated encephalopathy. **a** Representative images showing protein bands of Ki67, DCX, Nestin, BLBP, and GFAP from CLP mice injected with SOX2OT siRNA or scrambled control. Densitometry-based protein quantitation of **b** Ki67, **c** DCX, **d** Nestin, **e** BLBP, and **f** GFAP. β-actin was used as an internal control (*n* = 8/group). *p < 0.05; **p < 0.01. Abbreviations: CLP, cecal ligation and perforation; si-CRTL; scrambled control siRNA; si-SOX2OT, SOX2OT siRNA

CLP mice, with or without control siRNA, had significantly decreased protein expression of neuronal markers Ki67 (Fig. 6b), DCX (Fig. 6c), Nestin (Fig. 6d), and BLBP (Fig. 6e) in the hippocampus than the sham controls. Conversely, CLP animals showed 3-fold higher GFAP expression (Fig. 6f). Knockdown of SOX2OT in CLP mice partially normalized these effects of CLP, although expression levels remained significantly different from those in sham animals.

### SOX2OT knockdown decreased hippocampal SOX2 and OCT4 levels in mice with sepsis-associated encephalopathy

**Fig. 7:**
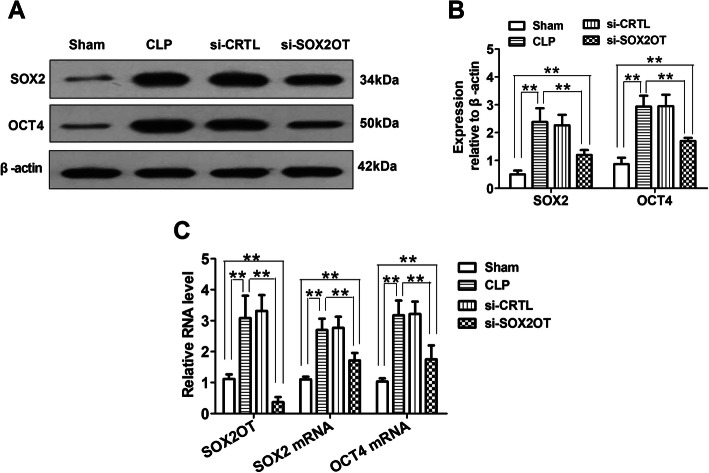
SOX2OT knockdown downregulated SOX2 and OCT4 in the hippocampus of mice with sepsis-associated encephalopathy. **a** Representative images showing SOX2 and OCT4 protein bands in hippocampal tissue from CLP mice injected with SOX2OT siRNA or scrambled control. **b** Densitometry quantitation of SOX2 and OCT4 expression. β-actin was used as an internal control. **c** Levels of SOX2OT, SOX2, and OCT4 transcripts (*n* = 8/group). **p < 0.01. Abbreviations: CLP, cecal ligation and perforation; si-CRTL, scrambled control siRNA; si-SOX2OT, SOX2OT siRNA

Western blot and RT-PCR assays showed that CLP mice expressed higher levels of SOX2 and OCT4 proteins (Fig. 7a, b) and transcripts (Fig. 7c) than sham mice. Treating CLP animals with SOX2OT siRNA downregulated SOX2 and OCT4 at the protein and mRNA levels. No significant difference was detected between CLP mice transfected with scrambled siRNA and untransfected CLP mice.

### SOX2 knockdown attenuated sepsis-induced cognitive dysfunction and changes in neuronal populations in mice with sepsis-associated encephalopathy

**Fig. 8:**
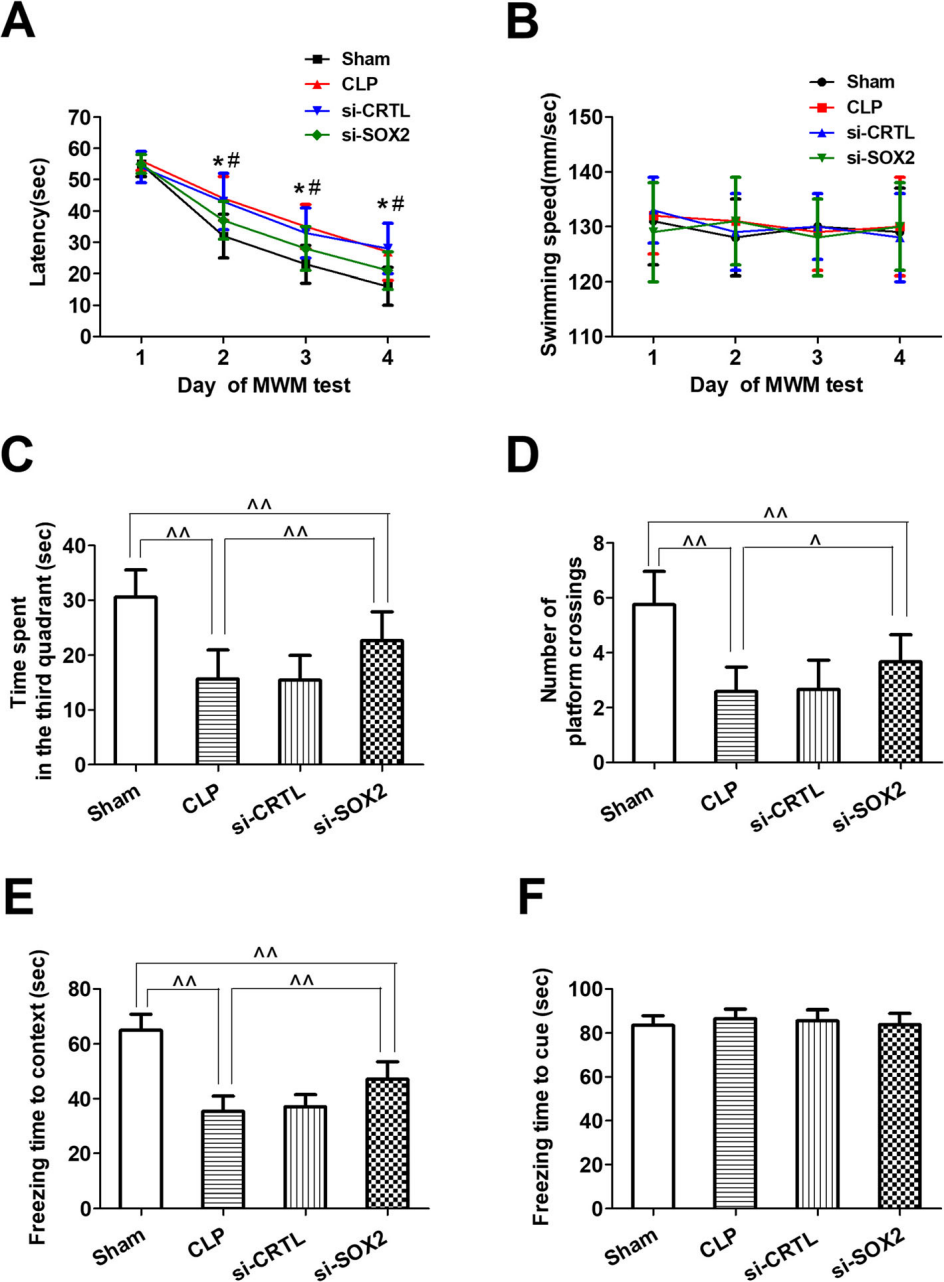
SOX2 knockdown using siRNA alleviated sepsis-induced cognitive dysfunction in sepsis-associated encephalopathy mice. **a** Escape latency, **b** swimming speed, **c** time spent in the target quadrant, and **d** number of times mice crossed the target quadrant on the test day in the Morris water maze. **e** Freezing time to context and **f** freezing time to cue in the fear conditioning test (*n* = 16/group). ^&^*p* < 0.05 vs. day 1 of Morris water maze test; *p < 0.05 vs. Sham; ^#^*p* < 0.05 vs. CLP; ^*p* < 0.05; ^^*p* < 0.01. Abbreviations: CLP, cecal ligation and perforation; si-CRTL, scrambled control siRNA; si-SOX2, SOX2 siRNA

**Fig. 9:**
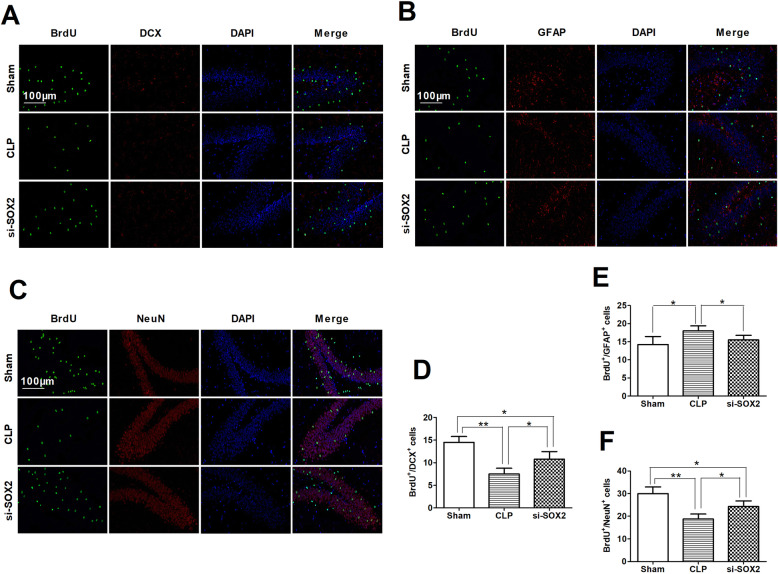
SOX2 siRNA attenuated the loss of cell proliferation and mature neurons and reduced glial differentiation in the hippocampus from sepsis-associated encephalopathy mice. Representative immunofluorescence micrographs showing (**a**) BrdU^+^/DCX^+^ cells, (**b**) BrdU^+^/GFAP^+^ glial cells and (**c**) BrdU^+^/NeuN^+^ neurons in hippocampal dentate gyrus of CLP mice injected with SOX2 siRNA. Magnification: × 200. Bar = 100 μm. Quantification of immunofluorescent cells staining positive for (**d**) BrdU/DCX, (**e**) BrdU/GFAP or (**f**) Brdu/NeuN. *p < 0.05; **p < 0.01. Abbreviations: CLP, cecal ligation and perforation; si-SOX2, SOX2 siRNA

SOX2OT somehow acts via SOX2 to repress neural progenitor proliferation and promote neuronal differentiation in the developing mouse cerebral cortex [15]. After observing that SOX2OT knockdown in our CLP mice downregulated SOX2 in hippocampal tissue, we asked whether knocking down SOX2 directly would have the same effects as knocking down SOX2OT. In the Morris water maze, SOX2 siRNA decreased escape latency in CLP mice (Fig. 8a), while there was no difference in swimming speed among groups (Fig. 8b). Similarly, SOX2 siRNA-transfected mice spent more time in and had more crossings of the target quadrant than untransfected CLP mice (Fig. 8c, d). SOX2 knockdown led to longer freezing time (Fig. 8e, f). Likewise, SOX2 knockdown was associated with increased levels of proliferation (Ki67) and maturation markers (NeuN) and decreased levels of differentiation (GFAP) (Fig. 6), and reversed CLP-induced changes in hippocampal neurogenesis (Fig. 9).

## Discussion

This study provides additional insights into the mechanisms behind cognitive deficits in sepsis-associated encephalopathy. First, our data demonstrated that sepsis induced by CLP surgery led to cognitive dysfunction and upregulation of SOX2OT and SOX2. Second, we found that SOX2OT knockdown inhibited downstream SOX2 expression and levels of neuronal markers in CLP mice, and reduced the number of cells positively stained for BrdU plus doublecortin and BrdU plus NeuN, except BrdU plus GFAP. Third, SOX2 knockdown produced similar effects as SOX2OT knockdown. Collectively, these data suggest that inhibiting the SOX2OT-SOX2 signaling axis can protect the hippocampus from sepsis-induced hippocampal neurogenesis deficits and neurodegeneration.

In this study, sepsis caused cognitive deficits that were detected as decreased time spent in the target quadrant in the Morris water maze test and shorter freezing time in a conditioned fear test. In addition, sepsis downregulated the expression of neuronal markers for stem cells (nestin and BLBP), proliferation (Ki67), and differentiation (DCX). Sepsis also led to higher levels of the astrocytic marker GFAP, indicating dysfunction differentiation of neurons in the hippocampal dentate gyrus. In many species, neural stem cells in the dentate gyrus of the adult hippocampus maintain the ability to generate self-renewing, multipotent neurons [6]. Direct or indirect stimulation of hippocampal neurogenesis, such as through exercise or consumption of green tea flavonoids, can enhance memory function and synaptic plasticity after neural stem cell differentiation [4, 7, 21]. However, aberrant proliferation and differentiation of adult neural stem cells or neuronal progenitor cells, induced by stress or pathological stimulation (e.g., through drugs, hormones, growth factors), can “derail” hippocampal neurogenesis, leading to deficits in cognitive functions including learning and memory [22–24]. For example, inducing sepsis in rats by CLP surgery disturbs adult neurogenesis in the subventricular zone [25]. Lipopolysaccharide-induced neuroinflammation in mice decreases immature and mature neuronal differentiation of neural stem cells and reduces neural stem cell proliferation and survival in adult hippocampal dentate gyrus [26]. Therefore, we speculate that sepsis-induced decline in learning and memory in CLP mice may be triggered by deficits in hippocampal neurogenesis.

Various lncRNAs have been shown to determine neural fate and regulate neurogenesis [9, 27]. SOX2OT is expressed in the developing mouse cerebral cortex, predominantly in the nucleus, and it represses neural progenitor proliferation and promotes neuronal differentiation [15]. Experiments in vitro have shown that SOX2OT is upregulated in the early stages of neural stem cell differentiation and then is gradually downregulated as differentiation proceeds [12]. During early neurodevelopment in mice, SOX2OT may regulate stemness, cell proliferation, and differentiation into neurons or glia [28]. We found that sepsis activated SOX2OT in the hippocampus, and SOX2OT knockdown attenuated sepsis-induced deficits in hippocampal neurogenesis and cognitive function. These results are consistent with previous results connecting SOX2OT upregulation with cognitive impairment in Alzheimer’s disease [13]. In addition, SOX2OT knockdown in adult mice can protect against hyperglycemia-induced retinal neurodegeneration [29].

Our results identified SOX2 as a downstream target of SOX2OT. Moreover, we found that SOX2 knockdown reduced sepsis-induced hippocampal neurogenesis and cognitive dysfunction, similar to the beneficial effects of SOX2OT knockdown. SOX2 is a transcription factor derived from a single exon within an intron of SOX2OT, and its coding sequence lies in the same transcriptional orientation as the lncRNA SOX2OT [12, 13]. SOX2 is uniformly expressed in neural stem cells and critical in the development, maintenance, and differentiation of hippocampal stem and progenitor cell populations [30–33]. Conditional ablation of SOX2 in adult neural progenitor cells impedes activation of pro-neural and neurogenic genes, resulting in increased neuroblast death and functionally aberrant newly derived neurons [34]. Our result is distinct from studies of SOX2 in the injured spinal cord injury [35], where it is found to show that resident astrocytes can be converted to DCX-positive neuroblasts by SOX2. Our results suggest that inhibition of the SOX2OT/SOX2 axis increases proliferation and survival of mature neurons, in a possible attempt to compensate for the devastating effect of sepsis and sepsis-associated encephalopathy on the brain.

Furthermore, our data suggest that sepsis upregulates the transcription factor OCT4 through the SOX2OT/SOX2 axis. OCT4 is the most studied SOX2-binding partner, and its overexpression in SOX2-null mouse embryonic stem cells rescues their pluripotency [36, 37]. This implies that SOX2 is crucial for maintaining their pluripotency, possibly through promoting and maintaining OCT4 expression.

There were some limitations to this study. We only examined brain neurogenesis in hippocampal dentate gyrus since the focus was sepsis-induced cognitive deficits. Brain neurogenesis can also be found in the subventricular zone (SVZ), which is an important source of neuronal regeneration [23–25]. The effect of sepsis on neurogenesis in SVZ remains unknown, warranting further investigation.

## Conclusions

In summary, in adult sepsis, the hippocampal SOX2OT are activated, resulting in deficits of hippocampal neurogenesis and cognitive function. SOX2OT knockdown attenuated sepsis-induced neurogenesis impairment and cognitive dysfunction, likely through its effects on downstream SOX2 signaling. We provide preliminary evidence that targeting SOX2OT/SOX2 axis may provide a therapeutic strategy for treating or preventing neurodegeneration in sepsis-associated encephalopathy.

## Data Availability

All data generated or analyzed during this study are available from the corresponding author upon request.

## Abbreviations

BLBP: Brain lipid-binding protein
BrdU: Bromodeoxyuridine
CLP: Cecal ligation and perforation
DAPI: 4,6-Diamidino-2-phenylindole
DCX: Doublecortin
GFAP: Glial fibrillary acidic protein
lncRNA: Long noncoding RNA
NeuN: Neuronal nuclei
OCT4: Octamer-binding transcription factor 4
PBS: Phosphate-buffered saline
RT-qPCR: Reverse transcription-quantitative polymerase chain reaction
SDS-PAGE: Sodium dodecyl sulfate–polyacrylamide gel electrophoresis
siRNA: Short interfering RNA
SOX2: Sex-determining region Y-box 2
SOX2OT: SOX2 overlapping transcript
SPSS: Statistical Package for Social Sciences
